# Detection of alterations in membrane metabolism during neoadjuvant chemotherapy in patients with breast cancer using phosphorus magnetic resonance spectroscopy at 7 Tesla

**DOI:** 10.1186/2193-1801-3-634

**Published:** 2014-10-27

**Authors:** Wybe JM van der Kemp, Bertine L Stehouwer, Peter R Luijten, Maurice AAJ van den Bosch, Dennis WJ Klomp

**Affiliations:** Department of Radiology, University Medical Center Utrecht, Heidelberglaan 100, 3485 CX Utrecht, The Netherlands

**Keywords:** Breast cancer, ^31^P MRSI, Neoadjuvant chemotherapy, Phosphomonoesters

## Abstract

Here we investigate the feasibility of tumor metabolism monitoring in T1c to T3 breast cancer during neoadjuvant chemotherapy by means of phosphorus (^31^P) magnetic resonance spectroscopy at 7 tesla (T). Five breast cancer patients were examined using a ^31^P MRSI sequence, prior to-, halfway-, and after neoadjuvant chemotherapy. The ^31^P MRSI data were analyzed on group and individual level and compared to a spectrum of a group of healthy volunteers. Ratios of phosphomonoesters (PME) to phosphodiesters (PDE) and phosphomonoesters to inorganic phosphate (Pi) were determined. Histopathologic assessment showed four partial responders and one complete responder to chemotherapy. The ^31^P spectrum of the patient group was distinctly different from the ^31^P spectrum of healthy volunteers and transformed its shape during the course of chemotherapy towards the shape of the spectrum of the healthy volunteers. Prior to chemotherapy the PME to PDE signal ratio and the PME to Pi signal ratio were high, and during the course of the chemotherapy these ratios normalized to the value of the healthy volunteers. Metabolite *T*_2_ values in tumor tissue tended to be lower than those for healthy glandular tissue. Assessment of individual patients showed that four out of five had a significant drop of the PME to Pi ratio by a factor of 2 or more. On average, the pH of the tumor, calculated from chemical shift variation of Pi, was 0.19 units lower before chemotherapy. We have demonstrated that the sensitivity of ^31^P MRSI in breast cancer at 7 T is sufficient to detect alterations in membrane metabolism during neoadjuvant chemotherapy, which may be used for early assessment of treatment efficacy.

## Introduction

Breast cancer is by far the most prevalent type of cancer in women worldwide (Jemal et al. 2011). Surgical breast cancer treatment is sometimes preceded by a neoadjuvant therapy such as chemotherapy, endocrine therapy, and or targeted therapy. Neoadjuvant therapy is applied in patients with inoperable inflammatory or locally advanced breast cancer and nowadays also in patients with operable breast cancer to reduce tumor volume to enable breast conserving surgery (Liu et al. 2010). Although reduction of the tumor volume is achieved in the majority of patients, a pathologic complete response is only seen in less than 20% of patients according to a recent meta-analyses which included almost 3800 breast cancer patients receiving neoadjuvant therapy (Kong et al. 2011). Because of this low pathologic complete response that is often used to predict survival rate, there is a need for early assessment of neoadjuvant chemotherapy efficacy, which would enable switching to a possibly more successful agent early in therapy. In contrast to imaging, that monitors the tumor’s morphology alterations during neoadjuvant therapy, the monitoring of tumor metabolism may offer a faster and better window to assess therapy efficacy. Phosphorus magnetic resonance spectroscopy (^31^P MRS) is probably the most suitable tool to achieve this, because the metabolites of interest are phosphorus compounds such as the phosphomonoesters (PME) phosphocholine (PC) and phosphoethanolamine (PE) and the phosphodiesters (PDE) glycerophosphocholine (GPC) and glycerophosphoethanolamine (GPE) that are involved in cell membrane anabolism and catabolism respectively (Podo 1999; Glunde et al. 2004). In addition to these metabolites, signals of mobile phospholipids glycerophosphatidylcholine (GPtC) and glycerophosphatidylethanolamine (GPtE), but also metabolites involved in energy metabolism such as inorganic phosphate (Pi) and adenosinetriphosphate (ATP) can be measured with ^31^P MRS.

Although hampered by a relatively low intrinsic sensitivity, ^31^P MRS offers substantial benefits above proton (^1^H) MRS in monitoring tumor metabolism. *In vivo* measurement of these individual ^31^P metabolites is feasible with ^31^P MRS, while *in vivo* proton MRS can only assess a ‘total-choline’ signal that comprises the sum of monoesters and diesters. In contrast to ^1^H MRS, that necessitates water suppression that may bias the total choline signal, phosphorous MRS doesn’t require water suppression. Moreover the chemical shift of Pi, as measured in ^31^P MRS may serve as a proxy for intracellular pH and tumor hypoxia.

The first clinical application of ^31^P MRS in monitoring neoadjuvant chemotherapy in breast cancer patients was reported in 1989 by Ng et al. (1989), after which a number of reports followed (Glaholm et al. 1989; Redmond et al. 1992; Twelves et al. 1994; Leach et al. 1998). All of these studies were performed at low magnetic field, having insufficient spectral resolution to resolve the individual phosphomonoester (PE, PC) signals or individual phosphodiester (GPE, GPC and mobile GPtE and GPtC) signals. Moreover, the sensitivity at these lower field strengths could only detect MR spectra from very large tumors, often involving almost the entire breast. Nonetheless, these previous studies report increased PME signal prior to therapy and decreasing PME during therapy for responders and partial responders. After the publication of Leach et al. (1998), it took 13 years to improve ^31^P MRS in breast cancer to facilitate clinical studies in more relevant tumor sizes. First of all, the field strength was increased from 1.5 T to 7 T, and efficient quadrature RF coils were designed dedicated to the human breast (Klomp et al. 2011). All together this improved sensitivity by an order of magnitude; a factor of 2 from 1.5 T to 3 T and more than a factor of 5 from 3 T to 7 T using the dedicated breast coil (Korteweg et al. 2011). Moreover, improved MRS sequences were developed that include spatial localization techniques and means to maximize sensitivity even further by using adiabatic multi-echo spectroscopic imaging (AMESING) (van der Kemp et al. 2013).

In this work we investigate whether state of the art ^31^P MRSI is sufficiently sensitive to pick up PME alterations in the primary tumor in patients with breast cancer during the course of neoadjuvant chemotherapy.

## Methods

### Patients

From the period February 2012 to August 2013, six patients diagnosed with breast cancer (one T1c, four T2 and one T3 stage cancer) who were scheduled for neoadjuvant chemotherapy at our institute were asked to participate in this study. The study protocol was approved by the Medical Ethics Committee of the Utrecht University Medical Center and all patients gave written informed consent. All patients received 6 doses of chemotherapy; either 6x FEC (fluorouracil + epirubicine + cyclophosphamide) or 3× FEC followed by 3× docetaxel. Patients underwent a ^31^P MRSI scan prior to-, half way- and after chemotherapy. Patients underwent surgery within a week after the last scan and chemotherapy efficacy was assessed by histopathology. This study is registered at trialregister.nl: NTR4652.

### MRI and MRSI

**Figure 1:**
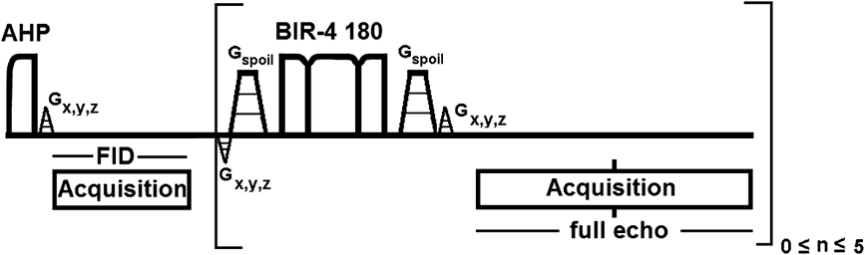
**Adiabatic multi-echo spectroscopic imaging (AMESING) sequence (van der Kemp et al.** 2013 **).**

Patients were scanned in prone position on a 7 T MR scanner (Philips Healthcare, Cleveland, OH, USA) using a unilateral two channel double tuned RF breast coil (MR Coils BV, Drunen, the Netherlands). The scan session contained a fat suppressed *T*_1_ weighted 3D MRI (*TE* =2 ms, *T*_R_ =4 ms, binominal 10 degree flip angle, FOV, resolution 1 mm^3^). *B*_0_-shimming was obtained over the breast using the standard Philips pencil beam shimming (Gruetter 1993). ^31^P MRSI was obtained using the AMESING sequence (Figure 1), in which 1 FID and 5 full echoes were acquired with Δ*TE* =45 ms; *T*_R_ =6 s; FOV 160×160×160 mm^3^; 8×8×8 voxels; (2 cm)^3^ nominal resolution; BW =8200 Hz; sampling matrix size =256; total scan time 25:36 min.

### Data analysis

Acquired data were zerofilled in the time domain and spatially Hamming filtered. The voxel containing the breast tumor was selected using 3DiCSI (Zhao et al. 2007) for visualisation. Spectra (1 FID and 5 full echoes) from tumor voxels of the patients, prior (P), halfway (HW), and after (A) neoadjuvant chemotherapy were frequency aligned for PE and weighted for the PE intensity of the FID to maximise SNR of the patient group spectra. The weighted sum FID spectra and the five weighted sum spectra of the full echoes, at the three points in time, were spectrally fitted in JMRUI (Naressi et al. 2001) using the AMARES (Vanhamme et al. 1997) algorithm. Average chemical shifts of the metabolites were calculated prior, halfway, and after neoadjuvant chemotherapy from FID and echo spectra. Prior to quantification the spectra were apodized with 40 Hz. During spectral fitting overall phases were fixed to zero, the linewidths of PE, PC and Pi were fixed to 55 Hz and the linewidth of GPtE + GPC in the FID was fixed to 65 Hz and in the echoes to 55 Hz (where predominantly GPC is left), the linewidth of GPtC was fixed to 65 Hz. The chemical shift of PE was kept in the range of 6.7 to 6.9 ppm, of GPtE + GPC in the range 2.7 to 2.9 ppm. Frequency differences between PE and PC, GPtE + GPC and GPtC, GPE and GPC were kept at 68 Hz. Metabolic signal ratios for PE/(GPtE + GPC), PC/GPtC, PME/PDE, and PME/Pi were calculated from spectral fitting. Weighted sum spectra of the patient group for the 5 full echoes were spectrally fitted as well and both FID and echo data were used to calculate metabolite *T*_2_ values. 95% Confidence intervals for the metabolite *T*_2_ values were calculated by means of Monte Carlo simulations.

Metabolic signal ratios for the individual patients during the course of neoadjuvant chemotherapy were calculated based on *T*_2_-weighting of the FID and the five symmetric echoes that were acquired for the tumor voxels. Metabolic *T*_2_-weighting was based on an assumed proportionality between accurately known average *T*_2_-values of ^31^P metabolites in healthy glandular tissue (Stehouwer et al. 2014) and those values in tumor tissue. To this end the ^31^P spectrum of a voxel containing the tumor was SNR optimized for all signals by fitting the same proportionality constant for all metabolites while applying the *T*_2_-weighting.

## Results and discussion

Within a time frame of 17 months, six patients participated in this study. One patient dropped out after the first scan session due to claustrophobia. Cancer type and receptor status of the breast cancers were assessed from core needle biopsies. Two patients of the group who successfully concluded this study were diagnosed with invasive ductolobular carcinoma, two with invasive ductal carcinoma, and one with medullary carcinoma. Three patients had receptor status ER +, PR +, Her2neu – and two were triple negative. Histopathologic assessment showed that the patient group that concluded the study (*n* =5) consisted of one full responder and four partial responders. After chemotherapy the full responder still had a fibroadenoma surrounding the tumor location.

**Figure 2:**
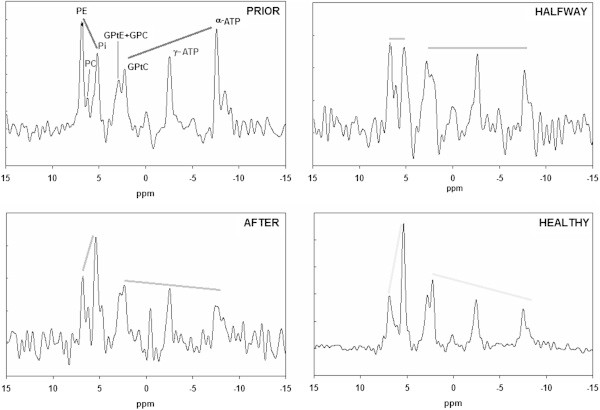
**Patient group FID spectra during neoadjuvant chemotherapy as compared to a FID spectrum of a group of healthy volunteers.** Data from healthy volunteers were obtained recently in our group by Stehouwer et al. (2014) and are based on 7 volunteers measured four times with four times larger voxel sizes leading to a high SNR group spectrum. The straight lines emphasize the change in metabolite signal intensities during therapy.

The FID spectra during neoadjuvant chemotherapy for the patient group are shown in Figure 2. For comparison, a FID spectrum of a healthy volunteer group consisting of seven healthy volunteers measured four times and with a four times larger nominal voxel size is shown as well (Stehouwer et al. 2014). There are a number of distinct features that distinguish the presented breast cancer spectra from the healthy volunteer group spectrum. These distinct features are most prominent when comparing the patient spectrum acquired prior to chemotherapy with the healthy volunteer group spectrum. Prior to chemotherapy the patient group spectrum shows higher: PE than Pi, PE than GPtE + GPC, and PE than GPtC. The signals of γ-ATP and α-ATP are higher than the PDE signals. In the healthy volunteer group spectrum theses patterns are reversed. During chemotherapy (half way and after chemotherapy) the patient group spectra are showing more resemblance to the healthy volunteer group spectrum.

**Figure 3:**
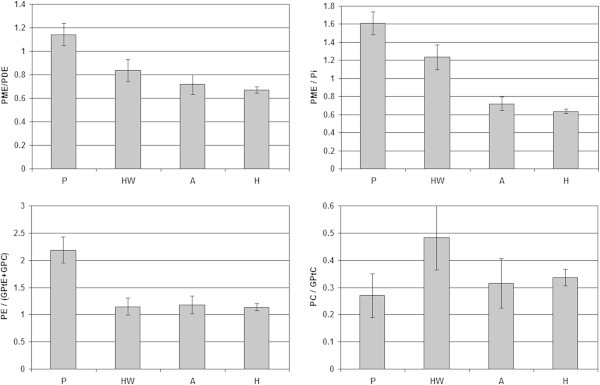
**PME and PDE metabolic signal ratios obtained by spectral fitting of FID group spectra of patients during neoadjuvant chemotherapy as compared to a group spectrum of 7 healthy volunteers.** P: Prior-; HW: Halfway-; A: After neoadjuvant chemotherapy; H: healthy.

In Figure 3 the PME to PDE signal ratios (PME/PDE, PE/(GPtE + GPC), PC/GPtC) and the PME to Pi metabolic signal ratios, as obtained from spectral fitting the FID group spectra, are shown during the course of chemotherapy, also in comparison to the healthy volunteer data by Stehouwer et al. (2014). Except for PC/GPtC, with the largest error bars, all metabolite signal ratios of the patient group PE/(GPtE + GPC), PME/PDE and PME/Pi show the same decreasing pattern during the course of chemotherapy and all normalize to values observed in healthy volunteers.

**Figure 4:**
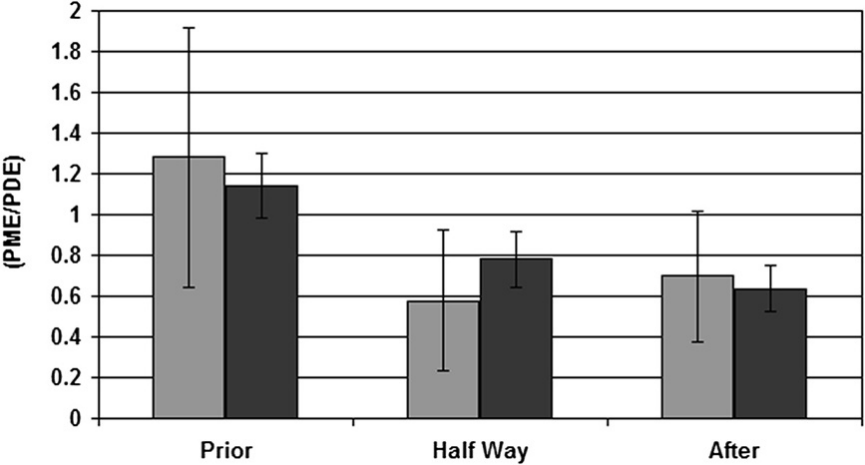
**Average PME over PDE signal ratios during the course of neoadjuvant chemotherapy calculated for individual patients (light grey, with physiological variation) and calculated from averaged patient group spectra (dark grey, without physiological variation).**

**Figure 5:**
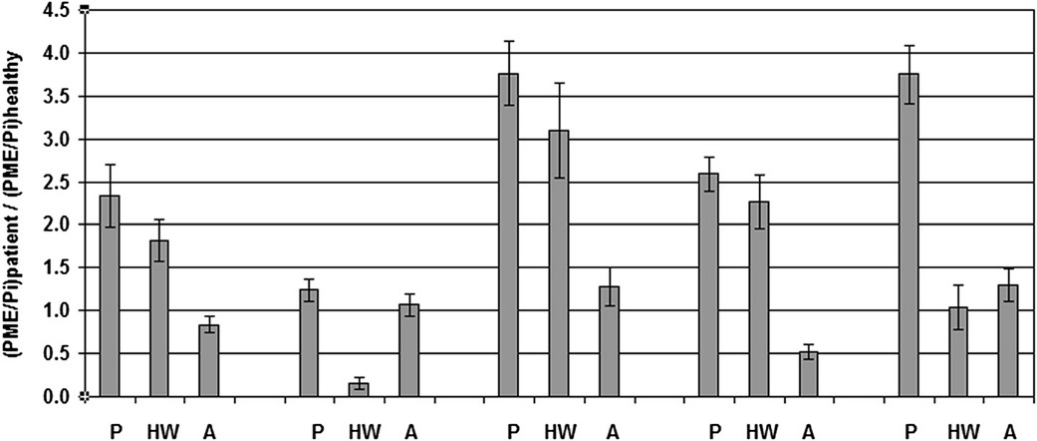
**Individual patient metabolic signal ratios of PME over Pi, during the course of chemotherapy, relative to healthy glandular tissue.** P: Prior-; HW: Half Way-; A: After neoadjuvant chemotherapy. For a value of 1.0 the PME over Pi signal ratio of patient equals that ratio in healthy glandular tissue. Note that all patients showed a higher PME over Pi ratio than 1.0 prior to therapy.

The metabolic signal ratios of the averaged group spectra shown in Figure 3 are based on average group signals (no physiological variability) and system noise (*i.e.* Cramer Rao lower bounds (De Graaf 2007) is represented by the error bars. In Figure 4 the PME over PDE ratios are shown as an average based on the individual patient data –including physiological variation as an error bar– and averaged group data with system noise as an error bar. The largest physiological variation between patients is seen in the PME over PDE ratios prior to chemotherapy as may be expected from this heterogeneous patient group with three ER+/PgR + and two triple negative patients. In preclinical work and animal studies it has been shown that triple negative breast cancers have a low PME to PDE ratio in contrast to ER+/PR + tumors which have a high PME to PDE ratio (Moestue et al. 2010; Esmaeili et al. 2014).Patient signal ratios of PME over Pi relative to healthy glandular tissue are shown during the course of chemotherapy, in Figure 5. Here, a ratio of 1.0 implies that patients PME over Pi signal ratio equals that of healthy glandular tissue.

Prior to neoadjuvant chemotherapy, four out of five patients show significantly higher PME over Pi signal ratios than encountered in healthy glandular tissue. After chemotherapy, one out of five patients shows a significantly lower PME over Pi signal ratio than encountered in healthy glandular tissue, while the other four patients have PME over Pi signal ratios similar to those in healthy glandular tissue. The PME over Pi ratio is a tissue viability marker that can be used in for instance assessing kidney quality for transplantation (Bretan et al. 1989), where a high ratio designates viable tissue. In radiation therapy a decrease of this ratio is an early marker for tumor cell apoptosis (Sakurai et al. 1998). However, pre-thermoradiotherapy PME to Pi ratios in sarcomas inversely correlate to pathologic complete response (Dewhirst et al. 2005).

**Figure 6:**
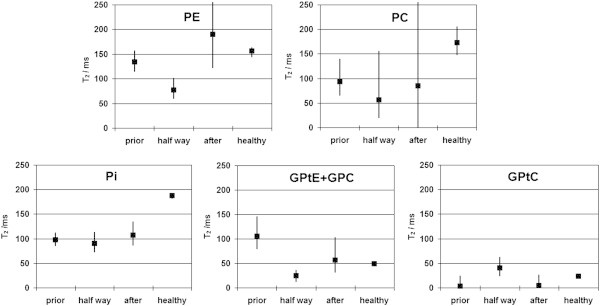
**Calculated metabolite** ***T***_**2**_ **values and 95% confidence intervals for several phosphorus containing metabolites in a breast cancer patient group during neoadjuvant chemotherapy.**

Calculated metabolite *T*_2_ values with 95% confidence intervals are shown in Figure 6 during chemotherapy and for healthy volunteers.

Metabolite *T*_2_ values fitted from low-SNR data are prone to be overestimated, as is evident from the asymmetric confidence intervals in Figure 6. Significantly lower *T*_2_ values than in healthy volunteers are seen for PE(HW), PC(P), Pi(P, HW, A) and GPtE + GPC(HW). Overall there is a trend for lower ^31^P metabolite *T*_2_ values in these breast cancer patients, as compared to the metabolite *T*_2_ values in healthy fibroglandular tissue. This matches the lower *T*_2_ values of water in prostate tumors as reported by Roebuck et al. (Roebuck et al. 2009). The significant higher *T*_2_ for the (GPtE + GPC) signal prior to chemotherapy is possibly caused by increased GPC prior to chemotherapy in this patient group, that includes two triple negative breast cancer patient cases. It is likely that GPtE has a *T*_2_-value similar to GPtC, *i.e.* in the order of 20 ms, while GPC is likely to have a *T*_2_ value that is in the range of the phosphomonoesters or even higher. In calf muscle for instance it exceeds 300 ms at 7 T (Bogner et al. 2009). This implies that on average the phosphodiester GPC signal (and concentration) is higher prior to chemotherapy and decreases during therapy in this patient group, which is contrary to the general, probably too simplistic, paradigm of increasing glycerophosphocholine and -ethanolamine during successful chemotherapy. This is corroborated by a report on the difference in choline metabolic profile of basal like and luminal like breast cancer xenografts by Moestue et al. (2010), where it was found that triple negative/basal like breast carcinomas had high GPC/PC ratios.

For the patient group as a whole the average chemical shift of Pi shows an increase of +0.14 ± 0.05 ppm over the course of the chemotherapy, which corresponds to a shift in pH of 0.19 units, implying a more acidic environment before the start of the chemotherapy, possibly indicative of tumor hypoxia or lactate formation.

In conclusion, using state of the art ^31^P MRSI provides sufficient sensitivity to detect statistically significant phospholipid alterations in primary breast tumors during neoadjuvant chemotherapy, despite the limited and heterogeneous sample size. Moreover, the ^31^P spectrum of the patient group obtained prior to treatment is distinctly different from the ^31^P spectrum of glandular tissue in the healthy volunteer group and transforms its shape during the course of the chemotherapy towards the shape of the spectrum of the healthy volunteer group. In line with our prospective hypothesis, the elevated phosphomonoesters in breast tumors decrease during the course of the chemotherapy. In addition, due to the high spectral resolution at 7 T, a subtle tumor pH increase of 0.19 units is observed during the treatment, possibly indicating tumor hypoxia or lactate formation.
